# Revisiting lupus low disease activity: validation matters, but vindication is imperative

**DOI:** 10.1093/rheumatology/keag196

**Published:** 2026-04-15

**Authors:** Ioannis Parodis

**Affiliations:** Division of Rheumatology, Department of Medicine Solna, Karolinska Institutet, Karolinska University Hospital, and Center for Molecular Medicine (CMM), Stockholm, Sweden; Department of Rheumatology, Faculty of Medicine and Health, Örebro University, Örebro, Sweden

**This editorial refers to the article ‘Validation of the revised definition of lupus low disease activity in patients with SLE’ by Ji-Hyoun Kang et al. 2026; 65(2), keag052.** 

In *Rheumatology*, Kang *et al.* [1] report a longitudinal validation of the revised lupus low disease activity state (LLDAS), aligned with the 2023 European Alliance of Associations for Rheumatology (EULAR) and the more recent American College of Rheumatology (ACR) recommendation that maintenance glucocorticoids in systemic lupus erythematosus (SLE) should not exceed a 5 mg/day prednisone equivalent [2, 3], rather than the previous 7.5 mg/day ceiling in the original LLDAS definition [4, 5]. In a Korean multicentre cohort of 299 patients followed annually over four years, attainment of the revised definition was significantly associated with lower damage accrual, lower flare risk, and better mental health-related quality of life [1]. Importantly, however, it did not outperform the original LLDAS definition, while fewer patients attained it. More precisely, this is a longitudinal validation of the revised EULAR-aligned definition rather than a study examining a ≤ 5 mg/day prednisone equivalent threshold *per se*; the feasibility of this has already been explored in prior work [6].

This is a timely and clinically relevant study. LLDAS has emerged as a pragmatic treat-to-target state in SLE, especially because remission, while aspirational, remains difficult to achieve and sustain in routine practice. Prospective and retrospective validation has shown that attainment of LLDAS is associated with protection from organ damage accrual and better health-related quality of life [7, 8], supporting its use as an end point in both observational studies and clinical trials. Against this background, revisiting the glucocorticoid threshold from 7.5 to 5 mg/day is not a trivial semantic change but a test of whether modern glucocorticoid stewardship can be integrated into an established target without sacrificing its clinical usefulness.

The main strength of the study is therefore conceptual as much as methodological: it addresses whether a stricter, safety-driven version of LLDAS remains clinically applicable. The answer appears to be yes. The revised definition was associated with lower SLICC/ACR Damage Index (SDI) scores, reduced flare risk, and improved SF-36 mental component scores [1].

What the study does not show, however, is that the revised definition is superior to the original one. On the contrary, the point estimates for protection against organ damage accrual and flares were numerically more favourable for the original LLDAS. The revised definition remained significantly associated with favourable outcomes, but being comparable should not be conflated with being superior, and this distinction matters if a revised target is to be promoted as the preferred treatment goal. In that sense, the present work supports validity but not a clear incremental benefit.

A second notable finding is the implementation gap. Annual attainment fell substantially when the stricter glucocorticoid threshold was applied, whereas sustained attainment over 4 years was only marginally different (27.8 vs 28.8%) [1]. This suggests that many patients who otherwise satisfy LLDAS remain just above the 5 mg/day threshold. That observation is clinically plausible. Therapeutic inertia, concern about relapse in patients with previous major organ involvement, withdrawal symptoms, and the non-specific symptomatic benefits patients may attribute to glucocorticoids can all impede tapering. The treatment profile of the cohort also suggests that this was largely a conventionally treated population, in which steroid-sparing may remain harder to operationalise than to recommend.

The study also prompts a broader question: how distinct is the revised LLDAS from remission? The DORIS definition of remission requires clinical SLEDAI-2K = 0, physician global assessment <0.5 (scale 0–3), prednisone or equivalent ≤5 mg/day, and stable background therapy [9]. The revised LLDAS is clearly less stringent than DORIS remission because it allows limited residual clinical activity, but the overlap has narrowed (Fig. 1). This is not necessarily a problem. In fact, the DORIS task force explicitly recommended that both remission and LLDAS may be used in clinical care and research, recognising that they serve related but different purposes [9]. Still, as the steroid threshold converges, future studies should clarify when the revised LLDAS identifies a clinically meaningful state distinct from near-remission, rather than simply a more attainable neighbour of DORIS remission. Moreover, as the therapeutic landscape in SLE evolves, expectations are rising and increasingly challenge existing definitions, pushing the field towards more stringent target states that are now becoming more attainable. In this context, even the contemporary definition of remission according to the DORIS criteria may eventually warrant reappraisal, particularly in view of the growing aspiration to withdraw glucocorticoids completely whenever possible [2, 3].

**Figure 1:**
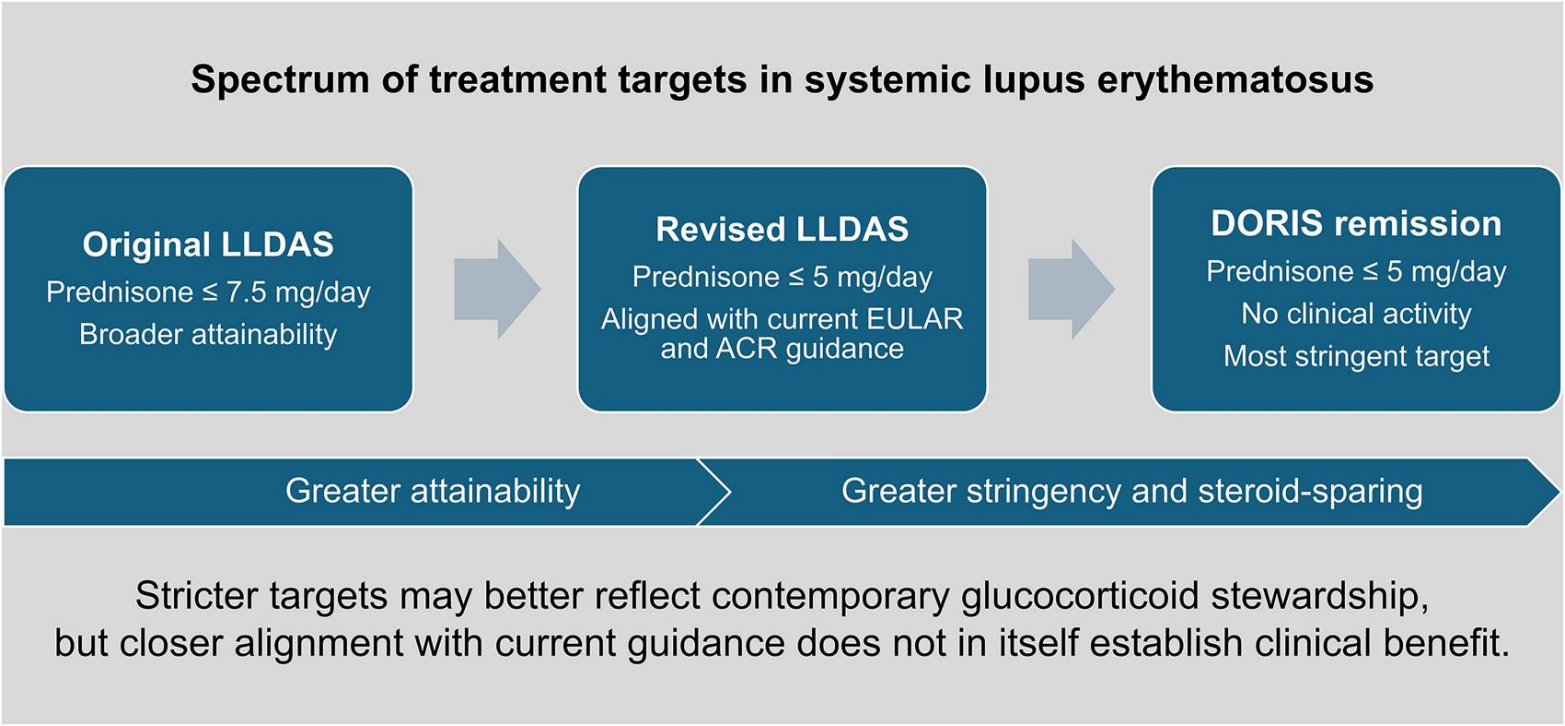
Conceptual relationship across original LLDAS, revised LLDAS, and DORIS remission in systemic lupus erythematosus. The original LLDAS definition permits a prednisone dose of up to 7.5 mg/day, whereas the revised LLDAS definition applies a stricter glucocorticoid threshold of ≤5 mg/day, in closer alignment with contemporary treatment recommendations. DORIS remission is a more stringent target state that also requires the absence of clinical disease activity. The figure illustrates the narrowing conceptual gap between revised LLDAS and remission, while emphasising that closer alignment with current guidance does not in itself establish superior clinical benefit. Abbreviations: ACR: American College of Rheumatology; DORIS: Definition of Remission in SLE; EULAR: European Alliance of Associations for Rheumatology; LLDAS: lupus low disease activity state; SLE: systemic lupus erythematosus.

There are, however, limitations that call for caution in interpretation. This was a single-country, ethnically homogeneous cohort, and glucocorticoid prescribing culture is known to vary internationally. The analyses were longitudinal, and the comparisons were based on annual attainment and summary outcomes, but methodologies incorporating more sensitive treat-to-target metrics, such as time to first flare, time-adjusted mean glucocorticoid exposure, or SDI trajectories, might have provided more nuanced insight. Another priority for future research is subgroup analyses in patients for whom glucocorticoid minimisation is especially challenging, such as those with lupus nephritis or neuropsychiatric SLE. Finally, studies specifically designed to examine whether the adoption of revised targets changes clinician behaviour would be highly welcome. From the present study by Kang *et al.*, one may conclude that it validates an updated definition, but successful implementation of such a strategy is yet to be demonstrated.

Overall, Kang *et al.* [1] provide an important first step. Their data suggest that tightening the glucocorticoid ceiling within LLDAS preserves its favourable prognostic utility and aligns the definition with current EULAR and ACR guidance. That is valuable. The next step is not simply to test whether the revised LLDAS can predict outcomes but whether using it prospectively can help clinicians taper steroids more effectively, without increasing flares, across diverse SLE populations. Given the broader literature linking glucocorticoid exposure to irreversible organ damage and multiple other adverse outcomes [10, 11], that question is not academic but central to long-term patient benefit. Validation has been achieved; vindication remains to be proven.

## Data Availability

No data were used in this article.
